# Androgen receptor reactivation in castration-resistant prostate cancer: mechanisms, epigenetic adaptation, and therapeutic vulnerabilities

**DOI:** 10.3389/fonc.2026.1837998

**Published:** 2026-05-21

**Authors:** Juntao Guo, Ke Wu, Zheng Ma, Shuai Guo, Fei Wang, Lingxiang Lu

**Affiliations:** 1 Department of Urology, Suzhou Ninth People’s Hospital, Soochow University, Suzhou, Jiangsu, China; 2 Department of Urology, The Affiliated Suzhou Hospital of Nanjing Medical University, Suzhou Municipal Hospital, Gusu School, Nanjing Medical University, Suzhou, Jiangsu, China

**Keywords:** androgen receptor reactivation, AR splicevariants, castration-resistant prostate cancer, epigenetic adaptation, precision therapy, therapeutic vulnerabilities, transcriptional reprogramming

## Abstract

Castration-resistant prostate cancer (CRPC) remains a difficult clinical problem, although androgen deprivation therapy and next-generation androgen receptor (AR) pathway inhibitors have greatly improved patient treatment. CRPC is not simply an androgen-independent disease. In many cases, tumor cells still depend on persistent or restored AR signaling under castrate conditions. AR reactivation is driven by several overlapping mechanisms, including AR amplification, AR overexpression, ligand-binding domain mutations, AR splice variants, intratumoral androgen synthesis, bypass signaling, and altered AR co-regulators. The AR axis is also connected with DNA damage repair. For example, PARP-1 can support both DNA repair and AR-driven transcription, which provides a rationale for combining AR-targeted therapy with PARP inhibition in selected patients. Epigenetic adaptation is another key layer in this process. Changes in chromatin accessibility, AR cistrome redistribution, pioneer factors, enhancer activity, and chromatin-modifying cofactors can reshape AR-dependent transcription. These changes help tumor cells maintain AR signaling and also promote heterogeneity, lineage plasticity, and more aggressive phenotypes. Recent single-cell transcriptomic and epigenomic studies further show that CRPC contains diverse resistant cell states, which may change during treatment. Importantly, these resistance mechanisms may also create therapeutic opportunities. Current and emerging strategies include AR degraders, AR N-terminal domain inhibitors, inhibitors of steroidogenesis and bypass pathways, PARP inhibitors, and epigenetic therapies targeting EZH2, BET proteins, p300/CBP, LSD1, or HDACs. Biomarker-guided treatment, including AR variants, DNA repair defects, ctDNA profiles, and chromatin states, may help select better therapies for CRPC patients.

## 1 Introduction

Prostate cancer is a hormone-dependent malignancy mainly driven by the androgen receptor (AR) signaling pathway (1, 2). For advanced disease, androgen deprivation therapy (ADT) alone, or in combination with next-generation AR pathway inhibitors (such as abiraterone and enzalutamide), remains the standard systemic treatment strategy (3). Although these treatments usually produce meaningful therapeutic responses, most tumors eventually progress to castration-resistant prostate cancer (CRPC) (4). Importantly, CRPC does not necessarily mean the tumor has entirely escaped regulation by androgen signaling. On the contrary, in many cases, persistent or reactivated AR activity remains a hallmark of disease progression (1).

This understanding has reshaped the perception of CRPC. CRPC is not simply an endocrine-independent state but is considered an adaptive state; in this state, tumor cells can maintain AR-driven transcriptional activity even when circulating androgens are deeply suppressed (5). AR reactivation can result from multiple overlapping mechanisms, including AR amplification, receptor overexpression, mutations in the ligand-binding domain, constitutively active AR splice variants, and intratumoral androgen biosynthesis. These changes collectively sustain transcriptional programs that support cell proliferation, survival, and treatment resistance, enabling tumors to continue progressing under ongoing therapeutic pressure (4, 5).

AR reactivation is also not entirely due to genomic alterations. Increasing evidence suggests that epigenetic adaptation can remodel chromatin accessibility, redistribute AR binding sites, and reprogram downstream transcriptional networks in CRPC (6). Through the coordinated actions of pioneer factors, chromatin-modifying factors, and transcriptional cofactors, tumor cells can stabilize AR signaling and acquire more plastic phenotypes, thereby promoting continuous evolution during therapy. This has important clinical implications, as it links AR reactivation to therapeutically targetable vulnerabilities (7).

Therefore, this article reviews the major molecular mechanisms of AR reactivation in CRPC, discusses how epigenetic adaptation reshapes AR-dependent transcription, and highlights emerging therapeutic vulnerabilities that may improve disease control in this increasingly heterogeneous and treatment-resistant disease context.

## 2 Molecular mechanisms of AR reactivation in CRPC

**Figure 1:**
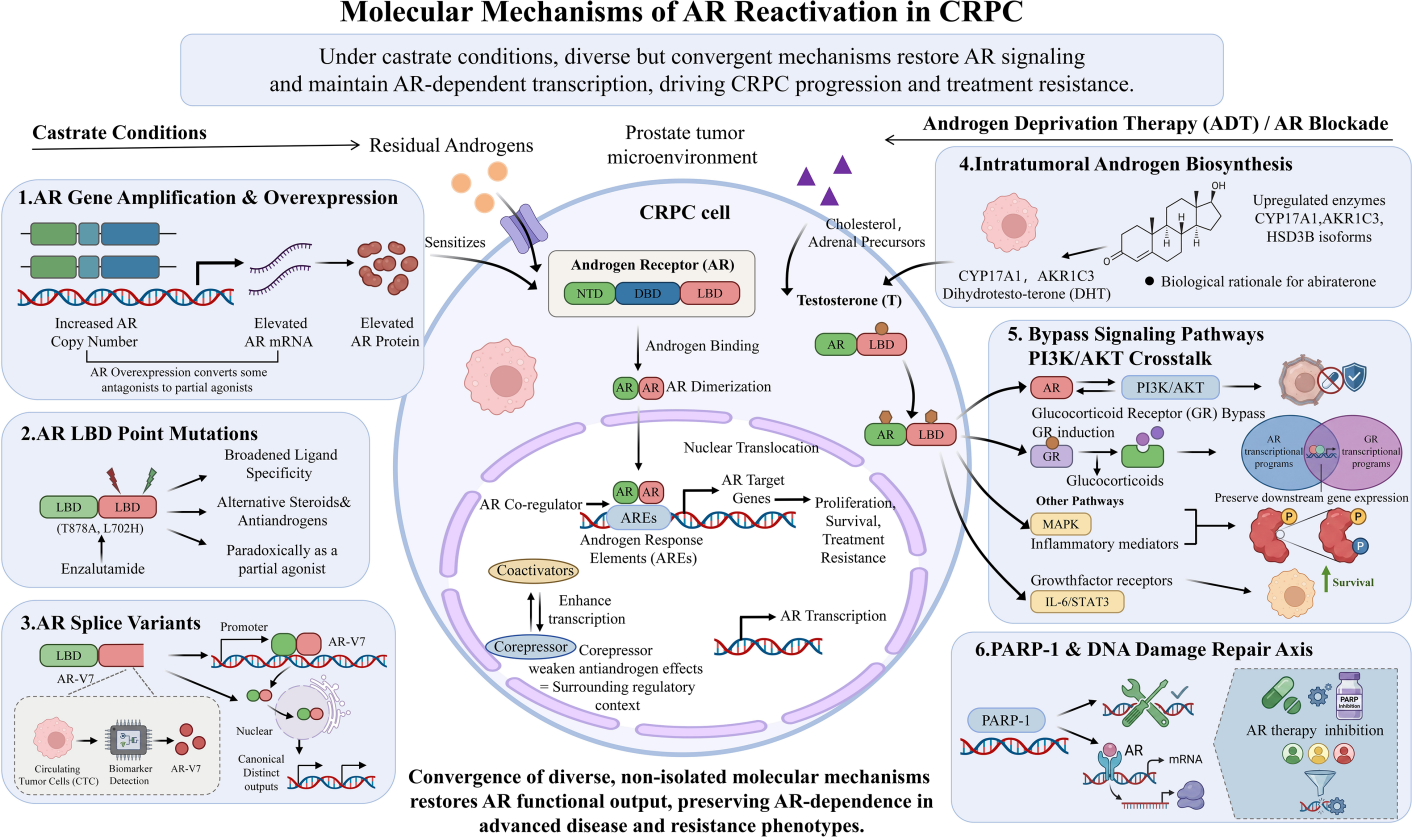
Molecular mechanisms of AR reactivation in CRPC. Schematic overview of the molecular mechanisms underlying androgen receptor (AR) reactivation in castration-resistant prostate cancer (CRPC). Under castrate conditions and selective pressure from androgen deprivation therapy (ADT) or AR blockade, CRPC cells restore AR signaling through multiple convergent mechanisms, including AR gene amplification and overexpression, point mutations in the AR ligand-binding domain (LBD), generation of AR splice variants, intratumoral androgen biosynthesis, bypass signaling pathways such as PI3K/AKT crosstalk and glucocorticoid receptor (GR)-mediated transcriptional programs, and PARP-1/DNA damage repair–associated regulation of AR transcription. These mechanisms enhance sensitivity to residual androgens, broaden ligand specificity, weaken antiandrogen efficacy, or directly sustain AR target gene expression, thereby promoting tumor cell proliferation, survival, and treatment resistance. The figure highlights that these processes are not isolated but converge within the prostate tumor microenvironment to preserve AR-dependent transcriptional output and drive advanced CRPC progression.

AR reactivation in CRPC originates from a diverse yet convergent set of molecular mechanisms that enable tumor cells to maintain AR-dependent transcription under castration conditions (8). These mechanisms do not act in isolation. Instead, they often coexist within the same tumor or occur sequentially during treatment, thereby collectively restoring functional output of the AR axis even when circulating androgens are suppressed (6). A core feature of this process is that CRPC cells become increasingly efficient at sensing, amplifying, or bypassing androgen input. One of the most well-defined mechanisms is AR gene amplification and overexpression (**Figure 1**).

Genomic gains of the AR locus are uncommon in untreated primary disease but become more frequent following ADT and AR-targeted therapy, suggesting a significant selective advantage under therapeutic pressure. Increased AR copy number is generally accompanied by elevated AR mRNA and protein expression, making tumor cells sensitive to very low androgen concentrations that would normally be insufficient to sustain signaling (9). In practical terms, this means that when receptor abundance is significantly increased, even residual adrenal androgens or low levels of intratumoral steroids may be sufficient to activate AR-dependent transcription. In some cases, AR overexpression may also convert certain antagonists into partial agonists, further promoting resistance (10).

The second major pathway involves AR point mutations, especially within the ligand-binding domain (LBD). These mutations can broaden ligand specificity, reduce antagonist efficacy, and even allow alternative steroids and anti-androgens to activate the receptor (11). As a result, therapies originally intended to inhibit AR signaling may maintain or reactivate receptor activity in mutated clones. Although not all CRPC cases carry these mutations, they are biologically significant because they indicate that selective therapeutic pressure can reshape the pharmacology of the receptor itself (12). LBD mutations are particularly relevant in advanced and heavily pretreated disease, as the cumulative effects of long-term endocrine therapy may promote expansion of resistant subclones with altered receptor function (12).

Among all AR reactivation mechanisms, AR splice variants have received particular attention because they provide a direct way to evade therapies targeting the receptor ligand-binding domain. The most extensively studied variant, AR-V7, lacks the classic LBD but retains the transcriptional activation domain and DNA-binding domain, enabling constitutive, ligand-independent transcriptional activity (13). Since drugs such as enzalutamide and abiraterone ultimately rely on inhibiting ligand-dependent AR signaling, the emergence of AR-V7 and related variants can reduce the effectiveness of these therapies (14). Importantly, AR splice variants do not simply replicate the activity of full-length AR. They can overlap with classical AR target genes while also promoting alternative transcriptional outputs associated with proliferation, survival, and therapy resistance (15). Therefore, detecting AR splice variants in circulating tumor cells has been explored as a clinically relevant biomarker of resistance in metastatic CRPC (16). However, routine clinical application of AR-V7 detection is still limited by heterogeneity in detection methods, including differences between circulating tumor cell-based detection and cell-free RNA liquid biopsy platforms, varying pre-analytical processing requirements, and inconsistent thresholds for defining AR-V7 positivity across studies (17, 18). These factors may limit comparability between studies and complicate the integration of AR-V7 status into standardized treatment decision-making.

Another key mechanism is intratumoral androgen biosynthesis, which allows CRPC cells to maintain sufficient local androgen levels even when systemic androgen production is suppressed (19). Advanced tumors are not entirely dependent on testicular androgen but may increasingly utilize adrenal precursors, cholesterol-derived substrates, and intracrine steroidogenesis pathways to generate testosterone or dihydrotestosterone within the tumor microenvironment (20). Enzymes such as CYP17A1, AKR1C3, and HSD3B isoforms are particularly important in this adaptive process (19). Their upregulation supports local steroid metabolism and maintains AR activation under androgen-depleted conditions. This mechanism helps explain why CRPC remains AR-driven despite seemingly effective castration and provides a biological rationale for therapies targeting steroidogenesis, including abiraterone and next-generation metabolic intervention strategies (16).

However, changes centered solely on the receptor cannot fully explain AR reactivation. Bypass signaling pathways and co-signaling networks also play important roles in restoring or maintaining AR function (20). Crosstalk between AR and the PI3K/AKT pathway is particularly significant because these two pathways can compensate for each other and promote cell survival under therapeutic stress. In some cases, inhibition of AR signaling enhances PI3K/AKT activity, while activation of this pathway can help stabilize a resistant phenotype (21). Similarly, MAPK signaling, inflammatory mediators, growth factor receptors, and cytokine-driven pathways such as IL-6/STAT3 are all believed to directly or indirectly support AR output. These pathways may enhance AR transcriptional activity, alter receptor phosphorylation status, or establish survival programs, thereby reducing cell dependence on classical androgen stimulation while retaining key AR regulatory functions (22).

The glucocorticoid receptor (GR), as part of a broader bypass mechanism, also merits attention (22, 23). GR can compensate for inhibited AR signaling by activating overlapping transcriptional programs, especially in the context of potent AR blockade. Although GR is not equivalent to AR, its induced expression indicates that CRPC cells can maintain key downstream gene expression patterns even when the primary receptor is pharmacologically suppressed (23). This phenomenon further increases therapeutic complexity because resistance may arise not only from AR recovery itself but also from activation of parallel nuclear receptor networks capable of sustaining relevant transcriptional outputs (23).

Moreover, AR co-regulators play a crucial role in determining the efficiency of receptor signaling and its consequences in CRPC (23). Co-activators such as members of the SRC family, p300/CBP, and other transcriptional partners can enhance AR-mediated transcription under low ligand conditions, whereas changes in the balance of co-repressors may weaken the inhibitory effects of anti-androgen therapy (6). These regulatory proteins help explain why similar levels of AR expression can result in different biological outcomes in different tumors. In other words, receptor abundance alone is insufficient to predict transcriptional strength; the surrounding co-regulatory environment largely influences whether AR signaling remains active and therapeutically relevant (23).

The AR signaling axis is also closely related to the DNA damage repair program, providing another therapeutic vulnerability for CRPC. Studies have shown that PARP-1 not only supports prostate cancer growth through its classical role in DNA repair, but can also act by promoting AR-driven transcriptional programs (24). This dual function provides a mechanistic rationale for combining AR-targeted therapy with PARP inhibition, particularly for metastatic CRPC tumors with homologous recombination repair defects (24, 25). Clinical and translational studies further support the importance of simultaneously targeting AR signaling and DNA repair pathways in advanced prostate cancer. For example, the combination of AR blockade and PARP inhibition has been explored as a strategy to exploit DNA repair dependency while suppressing AR-driven tumor growth (25). Additionally, genomic studies on metastatic prostate cancer have defined molecular features associated with homologous recombination DNA repair defects, reinforcing the importance of patient stratification when considering PARP inhibitor-based treatment regimens (26). Emerging research also suggests that AR-associated resistance programs may intersect with DNA repair and chromatin regulatory factors, including MECOM-dependent mechanisms in therapy-resistant prostate cancer (27). These observations indicate that the PARP/DNA repair axis should be regarded as an integrated component of AR-driven CRPC biology rather than an unrelated therapeutic topic.

In summary, these observations indicate that AR reactivation in CRPC is a multidimensional process driven by genomic amplification, receptor structural alterations, splice variant expression, adaptive steroid metabolism, and signaling pathway crosstalk (20). The convergence of these mechanisms helps explain the persistence of AR dependency in advanced disease and the remarkable heterogeneity observed in clinical resistance phenotypes. This molecular complexity also lays the groundwork for broader adaptation, where epigenetic remodeling can stabilize resistant transcriptional states and reshape the AR program itself (23).

## 3 Epigenetic adaptation and transcriptional reprogramming in CRPC

Genomic and metabolic changes alone cannot fully explain the remarkable plasticity exhibited by CRPC under continuous therapeutic pressure. Increasing evidence suggests that epigenetic adaptation plays a central role in maintaining, redirecting, and stabilizing AR signaling, even when androgen input is severely restricted (28, 29). In CRPC, the key issue is not only whether AR is present, but also how the chromatin landscape determines where AR binds, which genes it regulates, and how these outputs change during disease progression (29). Therefore, CRPC should be understood not only as a receptor-driven disease but also as a chromatin-adaptive disease.

A major feature of this process is chromatin remodeling and the redistribution of the AR cistrome (30). AR does not regulate the exact same set of target genes at all stages of prostate cancer. Under the pressure of ADT and AR pathway inhibitors, chromatin accessibility in tumor cells undergoes widespread changes, forming new regulatory regions and altering enhancer usage, causing AR to occupy a set of genomic binding sites different from those seen in the treatment-naive disease (31, 32). This shift allows CRPC cells, while retaining AR dependency, to adopt transcriptional programs better suited for survival in a castrate environment (32). Therefore, AR signaling is not simply restored but functionally reprogrammed.

This reprogramming is strongly influenced by pioneer factors and lineage-specific transcription factors, especially FOXA1, HOXB13, and GATA2 (33). These proteins can open chromatin, define lineage-specific regulatory elements, and guide AR to specific sites (7). In CRPC, changes in their expression or activity promote AR occupancy patterns favorable for growth under resistance conditions. For example, FOXA1 can facilitate AR access to previously inaccessible regions, while HOXB13 is considered a critical determinant of late-stage AR signaling programs, including those associated with AR splice variant activity (7). Together, these factors provide a scaffold that allows AR signaling to continue evolving without a complete loss of receptor dependency.

Meanwhile, epigenetic cofactors and chromatin-modifying enzymes further reinforce these adaptive states (29). EZH2 has drawn widespread attention for its role in CRPC that goes beyond classical Polycomb-mediated repression (34). In advanced disease, EZH2 may support oncogenic transcription and lineage plasticity in noncanonical ways and, in some cases, synergize with AR-related programs (35). BRD4 and other BET proteins also contribute to maintaining transcriptionally active chromatin at enhancers and super-enhancers associated with disease progression (32). By linking histone acetylation to transcriptional elongation, they sustain AR-centered or AR-proximal programs under therapeutic pressure (36).

Other regulators, including p300/CBP, HDACs, LSD1, and other lysine demethylases, further shape the intensity and specificity of AR-dependent transcription (37). p300/CBP enhances transcription through histone acetylation and coactivator function, while HDACs and LSD1 have context-dependent effects on chromatin structure and gene expression (38). Their importance lies in creating a permissive epigenetic environment for adaptive signaling, rather than simply acting as a switch.

Epigenetic adaptation also promotes lineage plasticity, allowing CRPC cells to move from a highly AR-dependent adenocarcinoma state to a weakly AR-dependent or even AR-independent phenotype along a continuous spectrum (39). This shift may occur gradually. The same flexibility that initially helps maintain AR reactivation may also subsequently freed cells from AR dependence (39). Therefore, AR reactivation and lineage plasticity are not always opposing processes, but may represent different outputs produced by a co-adaptive system driven by therapeutic stress and chromatin remodeling (30).

Recent advances in single-cell transcriptomics and epigenomics have further deepened our understanding of this adaptation process (40, 41). Although holistic tumor lineage analysis has provided important insights into recurrent genomic and epigenetic alterations in CRPC, it may mask cellular heterogeneity and transition states that occur under treatment stress (42). Single-cell RNA sequencing enables the identification of different tumor subpopulations, resistant cell statuses, and lineage transition phenotypes that may coexist in the same lesion (43, 44). At the same time, single-cell ATAC sequencing can map chromatin accessibility at high resolution, revealing how enhancer use, transcription factor activity, and AR-related regulatory programs are remodeled in a single cell (40). Together, scRNA-seq and scATAC-seq provide a powerful framework for reconstructing lineage trajectories during treatment, helping to distinguish between persistent AR-driven states and weakly AR-dependent or AR-independent phenotypes, and elucidating the mode of remodeling of AR cis-regulators during disease progression to treatment-resistant states (40, 45, 46). Therefore, the inclusion of these methods in CRPC studies is crucial for linking epigenetic adaptations to intratumoral heterogeneity, lineage plasticity, and treatment resistance evolution.

Overall, epigenetic adaptation does not occur only with AR reactivation in CRPC; It actively reshapes the transcriptional landscape, making AR signals persistent, flexible, and clinically important (29). By reshaping chromatin accessibility, redirecting AR binding, and stabilizing drug-resistant cell status, CRPC cells translate AR signaling into a dynamic and evolving regulatory network. This recognition has important therapeutic implications because key therapeutic vulnerabilities may be present not only in the receptor itself, but also in the epigenetic mechanisms that maintain its adaptive output (34).

## 4 Therapeutic vulnerabilities and emerging treatment strategies

Key aging-associated immune alterations, their mechanisms, relevance to prostate cancer, and potential therapeutic implications are summarized in **Table 1**. Adaptive changes that enable tumors to survive under endocrine treatment pressure may also produce specific therapeutic vulnerabilities, especially when CRPC still relies on AR signaling, adaptive steroid metabolism, or epigenetic mechanisms to maintain a resistant transcriptional state (39). Therefore, the challenge is not just to further enhance androgen suppression, but to identify which part of the adaptive AR network is most critical in specific tumors (47, 48).

**Table 1:** Therapeutic vulnerabilities and emerging treatment strategies in CRPC.

Therapeutic category	Representative targets/approaches	Representative agents/clinical trials	Mechanistic rationale	Clinical implication
AR-axis suppression or dismantling	Next-generation AR inhibitors; PROTAC-based AR degraders; N-terminal domain inhibitors	Bavdegalutamide (ARV-110; PROTAC AR degrader, phase 1/2, NCT03888612); masofaniten/EPI-7386 (AR NTD inhibitor, phase 1/2 or phase II combination studies); EPI-506 (first-generation AR NTD inhibitor, phase I) (68–70)	Targets persistent AR dependence more directly than conventional ligand-binding blockade and may overcome resistance associated with AR overexpression, altered receptor pharmacology, and AR splice variants such as AR-V7.	Most relevant in tumors that remain strongly AR-driven despite prior exposure to abiraterone or enzalutamide, especially those with AR amplification, LBD alterations, or splice variant-driven resistance.
Adaptive steroidogenesis targeting	AKR1C3 and related steroidogenic enzymes; inhibition of intratumoral androgen biosynthesis	Indomethacin-based AKR1C3 inhibition strategies; investigational AKR1C3-directed agents; abiraterone as an established steroidogenesis-targeting backbone (2, 71)	Reduces local androgen availability under systemic castration and may restore sensitivity to AR-directed therapy.	Applicable when intracrine androgen production or steroidogenic enzyme upregulation is a dominant resistance mechanism.
Bypass pathway co-targeting	PI3K/AKT, MAPK, and glucocorticoid receptor signaling	Ipatasertib plus abiraterone in IPATential150, phase III; mifepristone plus enzalutamide as dual AR/GR antagonism, phase I/II; other selective GR modulators under early clinical or translational development (59, 60, 72)	Interrupts compensatory signaling networks that support the survival of resistant clones and maintain AR-related transcriptional output despite pharmacologic AR inhibition.	Combination strategies are attractive because reciprocal feedback between AR and PI3K/AKT signaling, as well as GR-mediated bypass signaling, may limit the efficacy of single-pathway inhibition.
Epigenetic vulnerability targeting	EZH2 inhibitors; BET inhibitors; p300/CBP inhibitors; LSD1/HDAC-directed approaches	EZH2 inhibitors, including mevrometostat/PF-06821497 and tazemetostat-based combinations; BET inhibition with ZEN-3694 plus enzalutamide; and p300/CBP inhibition with CCS1477/inobrodib (36, 73, 74)	Disrupts the chromatin-based maintenance of adaptive AR signaling, resistant transcriptional programs, enhancer-driven oncogenic transcription, and lineage plasticity.	Best suited for combination with AR-directed therapy to weaken the transcriptional environment that sustains resistance, particularly in tumors with epigenetic reprogramming or lineage-plastic features.
Biomarker-guided precision treatment	AR amplification; AR splice variants such as AR-V7; ctDNA profiles; steroidogenic and chromatin states	AR-V7 circulating tumor cell assays; ctDNA-based detection of AR alterations, DNA repair defects, PTEN loss, TP53/RB1 loss, and chromatin-regulatory signatures; longitudinal liquid biopsy monitoring (15, 18, 75, 76)	Matches therapy to the dominant resistance mechanism in an individual tumor and supports longitudinal monitoring during sequential treatment.	Important for patient stratification, treatment selection, adaptive trial design, and real-time modification of therapeutic strategy.

AR, androgen receptor; CRPC, castration-resistant prostate cancer; ctDNA, circulating tumor DNA; PROTAC, proteolysis-targeting chimera.

A major strategy is to improve the inhibition or degradation of the AR axis itself (48). Although abiraterone and enzalutamide have already changed the therapeutic landscape, their efficacy is still limited by cross-resistance as well as the eventual emergence of ligand-independent or highly sensitive AR states (49). This has driven interest in AR degraders and other next-generation inhibitors that go beyond classic ligand-binding blockade. For example, PROTAC-based AR degraders may overcome resistance associated with AR overexpression or pharmacologic changes, as they reduce receptor abundance rather than merely inhibiting receptor activation (50, 51). Compounds targeting the N-terminal domain are also attractive because this region is present in both full-length AR and many constitutively active splice variants, including AR-V7 (51, 52).

In this context, targeting the AR N-terminal domain (NTD) has become a particularly important therapeutic strategy (53). Unlike traditional anti-androgens that bind the ligand-binding domain (LBD), NTD-targeting drugs aim to inhibit AR transcriptional activity through a region retained in both full-length AR and many constitutively active AR splice variants, including AR-V7 (51, 52). This feature makes NTD inhibition especially appealing for tumors that evade LBD-targeted therapy via splice variant expression or ligand-independent AR activation (54). Early NTD inhibitors, including the EPI series of compounds, provided proof-of-concept that pharmacological interference with AR transactivation can suppress AR-driven transcription even in the absence of a complete LBD (51). Recent small-molecule NTD inhibitors have further refined this strategy, showing greater potency, better drug-like properties, and activity against AR splice variant-driven models (55, 56). These drugs represent a mechanistically distinct class of AR-targeted therapies, as they are designed to inactivate the transcriptional core of AR signaling, rather than compete for ligand binding (57). Therefore, expanding discussion of NTD-targeting small molecules is critical for understanding how future treatments might overcome resistance mediated by AR-V7 and related splice variants.

The second opportunity lies in targeting adaptive steroidogenesis and bypass pathways. Since intratumoral androgen biosynthesis contributes to maintaining AR signaling under systemic castration, enzymes such as AKR1C3 and related steroidogenic components are rational therapeutic targets (19, 20, 52). Blocking these metabolic nodes may reduce local androgen availability and restore sensitivity to AR-targeted therapy. Meanwhile, bypass pathways such as PI3K/AKT, MAPK, and glucocorticoid receptor signaling provide additional intervention points, as they support resistant clones and may compensate for AR inhibition (58). Given the reciprocal feedback between AR and the PI3K/AKT pathway, combined strategies targeting both AR and PI3K/AKT are particularly attractive (58).

In the context of potent AR blockade, the glucocorticoid receptor (GR) is especially relevant (22). GR can activate partially overlapping transcriptional programs, thereby substituting for AR activity in resistant tumor cells. This compensatory mechanism provides a rationale for therapeutic strategies aimed at inhibiting or modulating GR signaling (59). Selective GR antagonists, including mifepristone and more selective non-steroidal GR modulators, have been explored as potential approaches to prevent or reverse GR-mediated resistance (59, 60). In principle, combining GR antagonism with AR pathway inhibition could simultaneously suppress the primary AR programs and the parallel nuclear receptor networks that arise under therapeutic pressure (22, 60). However, since GR also regulates normal stress responses, metabolism, and immune function, future strategies must balance antitumor efficacy with systemic toxicity (61). Biomarker-guided selection of patients with GR upregulation or GR-dependent transcriptional states may therefore be important for the clinical development of GR-targeted combinations in CRPC.

A particularly important vulnerability is the dependence of CRPC on epigenetic support systems that stabilize adaptive AR signaling. If chromatin remodeling and transcriptional reprogramming are essential for resistant AR output, then the proteins that maintain these states become druggable liabilities (62). This rationale has driven interest in EZH2 inhibitors, BET inhibitors, p300/CBP inhibitors, and agents targeting LSD1, HDACs, or related chromatin regulators (36, 62). These agents are especially attractive in combination with AR-directed therapy, because they may disrupt the transcriptional environment that allows tumor cells to preserve AR function under treatment pressure. Inhibition of BET proteins or p300/CBP may weaken enhancer-driven oncogenic transcription, whereas EZH2 inhibition may interfere with lineage plasticity and resistant cell-state maintenance (37).

Epigenetic therapies may also be relevant to more extreme forms of lineage plasticity, in which prostate cancer cells transition toward weakly AR-dependent or AR-indifferent phenotypes. Clinically, these states include treatment-induced neuroendocrine prostate cancer (t-NEPC) and double-negative prostate cancer (DNPC). t-NEPC is characterized by the emergence of neuroendocrine differentiation, decreased dependence on canonical AR signaling, and aggressive clinical behavior after exposure to potent AR pathway inhibitors. In contrast, DNPC lacks both AR signaling and neuroendocrine marker expression, representing another AR-independent resistant state (63, 64). These phenotypes suggest that resistance can progress from AR reactivation to lineage reprogramming and loss of prostate epithelial identity. Mechanistically, this extreme plasticity is often associated with concurrent genomic deletion or functional inactivation of TP53 and RB1, accompanied by epigenetic remodeling driven by chromatin regulators and lineage-determining transcription factors (65, 66). Therefore, therapies targeting EZH2, BET proteins, LSD1, or other epigenetic dependencies may be particularly suitable for tumors transitioning toward t-NEPC, DNPC, or other states of lineage plasticity (67).

These strategies also highlight the necessity of biomarker-guided precision treatment. Because CRPC is molecularly heterogeneous, not all patients will benefit equally from the same intervention. Biomarkers such as AR amplification, AR splice variant expression, circulating tumor DNA profiles, steroidogenic enzyme upregulation, and broader transcriptional or chromatin states may help define the dominant resistance mechanism in an individual tumor (5). AR-V7 is one of the best-known examples of a clinically informative biomarker associated with resistance to certain AR pathway inhibitors (15, 16). More broadly, longitudinal molecular monitoring may become increasingly important as CRPC evolves under sequential therapy (48).

Taken together, the most promising treatment strategies for CRPC are likely to be those that address adaptive dependence rather than a single static pathway (49). Combining AR-directed therapies with interventions against steroidogenesis, bypass signaling, or chromatin-based maintenance mechanisms - while using biomarkers to stratify patients - may yield more durable benefit (5).

## 5 Discussion and future directions

AR reactivation remains a central feature of CRPC after ADT and next-generation AR pathway inhibitors. However, this process is not caused by a single alteration. AR amplification, receptor mutation, splice variants, intratumoral androgen synthesis, bypass signaling, and epigenetic remodeling can all contribute to persistent AR activity. These mechanisms often coexist and change during treatment, which partly explains the strong heterogeneity of CRPC and the different responses among patients. Another important point is that CRPC is not only an AR-driven disease, but also an epigenetically adaptive disease. Chromatin remodeling, enhancer rewiring, pioneer factors, and chromatin-modifying enzymes can reshape AR-dependent transcription under therapeutic pressure. These changes help tumor cells maintain AR signaling in a low-androgen environment. At the same time, they may also promote lineage plasticity and drive some tumors toward weakly AR-dependent or AR-indifferent phenotypes, such as t-NEPC and DNPC.

Therefore, future treatment should not simply rely on stronger AR blockade. More effective strategies may need to combine AR-targeted therapy with approaches against steroidogenesis, bypass pathways, DNA repair, and epigenetic regulators. AR degraders, AR NTD inhibitors, PARP inhibitor-based combinations, GR modulation, and EZH2, BET, p300/CBP or LSD1-targeted therapies are all worth further investigation.

Finally, biomarker-guided treatment will be very important. AR alterations, AR splice variants, DNA repair defects, ctDNA profiles, GR activity, and chromatin states may help identify the dominant resistance mechanism in each patient. Because CRPC evolves during sequential therapy, longitudinal monitoring is also needed. A better understanding of the dynamic relationship among AR reactivation, epigenetic adaptation, and lineage plasticity may help design more rational combinations and improve the management of advanced CRPC.
